# Bridging the Gap: A Narrative Review of the Essential Role of Occupational Therapists in Sensory Stimulation for Comatose Traumatic Brain Injury Patients

**DOI:** 10.7759/cureus.108338

**Published:** 2026-05-06

**Authors:** Ahmad Alqasem, Aya Odeh

**Affiliations:** 1 Occupational Therapy, Jordan University of Science and Technology, Irbid, JOR; 2 Pediatrics, Royal Jordanian Medical Services, Amman, JOR

**Keywords:** coma recovery, comatose patients, disorders of consciousness (doc), multimodal stimulation, occupational therapy, sensory stimulation, traumatic brain injury (tbi)

## Abstract

Traumatic brain injury (TBI) frequently results in prolonged disorders of consciousness (DOC). Although sensory stimulation is a recognized early intervention, the specific contribution of occupational therapists (OTs) remains underexplored. This narrative review evaluates the efficacy of sensory stimulation in patients with TBI-related DOC, with a focus on comparing OT-led interventions with those delivered by other caregivers. A narrative review of 13 studies published between 2015 and 2025 was conducted, examining interventions across acute and rehabilitation settings involving various sensory modalities. Findings suggest that sensory stimulation, regardless of provider, may improve levels of consciousness as measured by the Glasgow Coma Scale (GCS) and Coma Recovery Scale-Revised (CRS-R). Limited evidence from a single OT-led study indicates the potential for additional functional gains associated with structured and individualized multimodal sensory stimulation. While non-specialized stimulation appears effective in enhancing basic arousal, occupational therapy may contribute through a more graded, individualized approach that supports emerging functional responsiveness. Integrating occupational therapy expertise into sensory stimulation protocols may contribute to improving the quality and functional relevance of interventions. However, current evidence remains limited, and future research should prioritize well-designed studies to develop and validate standardized OT-led multimodal protocols and establish clearer clinical guidelines.

## Introduction and background

Traumatic brain injury (TBI) is a significant global public health concern, often resulting from external physical forces that disrupt normal brain function and lead to prolonged disorders of consciousness (DOC). Severe TBI is associated with profound cognitive, motor, sensory, and emotional impairments that pose substantial challenges for recovery and quality of life. Early and targeted rehabilitation is critical, as persistent disorders of consciousness or delayed emergence can adversely affect long-term outcomes and functional potential [1,2].

From a clinical perspective, DOC encompasses a spectrum of states ranging from unresponsive wakefulness syndrome (UWS), characterized by wakefulness without awareness, to minimally conscious state (MCS), where patients demonstrate inconsistent but reproducible signs of awareness [1,2]. The transition between these stages is particularly important for rehabilitation, as emerging purposeful responses indicate increased neurobehavioral readiness for structured sensory stimulation and engagement [2].

Sensory stimulation is an intervention designed to enhance alertness and behavioural responsiveness by applying targeted environmental stimuli in patients with DOC [2]. Originally proposed as an early neurorehabilitation strategy for patients with severe TBI, its underlying principle is that controlled and structured sensory input delivered through auditory, visual, tactile, olfactory, gustatory, or multimodal pathways may support neuroplasticity and cortical arousal [3].

Despite this potential, current evidence remains heterogeneous, with considerable variation in stimulation protocols, outcome measures, and intervention providers, including nurses, family members, and therapists. Although a substantial body of research supports the use of sensory stimulation for DOC, the explicit role of occupational therapists (OTs) appears to be less represented in many published studies and intervention protocols. This persists despite OTs possessing specific expertise in sensory regulation and the provision of individualized, meaningful stimulation tailored to patient preferences-an important factor in optimizing neurorehabilitation outcomes [2,4]. While broader rehabilitation literature suggests that OT involvement may be associated with improved cognitive and motor outcomes and faster recovery [5], the specific contribution of OT-led structured sensory stimulation approaches requires further investigation.

Within this context, occupational therapy may be particularly relevant during the transition from UWS to MCS, when patients begin to demonstrate inconsistent but meaningful responses requiring careful observation and graded sensory input. OTs are well-positioned to adjust the intensity and complexity of stimulation to support emerging awareness while minimizing the risk of overstimulation, consistent with principles of sensory integration and individualized neurorehabilitation approaches [5].

Given the complexity of DOC and the need for early, individualized intervention, this narrative review synthesizes recent research on sensory stimulation in comatose TBI patients with a specific focus on the role of occupational therapy. By comparing studies involving different intervention providers and modalities, this review aims to explore the potential contribution of OT involvement within sensory stimulation programs and identify directions for future research, highlighting OT-informed approaches as a potentially valuable component of multidisciplinary neurorehabilitation.

Method

Study Design and Search Strategy

 This narrative review examined the role of OTs in enhancing consciousness in adult patients with comatose states following TBI. A literature search was conducted using PubMed and Google Scholar to identify relevant studies published in English between 2015 and 2025. Search terms included "coma," "sensory stimulation," "traumatic brain injury," "consciousness level," "cognitive function," and "occupational therapy." The initial search resulted in 327 articles. After screening for eligibility based on population, intervention, outcomes, and study design, 20 articles were included for full-text review.

PubMed was used for its broad coverage of biomedical and rehabilitation literature, while Google Scholar supplemented search sensitivity by capturing additional peer-reviewed and relevant grey literature. Although databases such as CINAHL (Cumulative Index to Nursing and Allied Health Literature) and the Cochrane Library were not included, the selected sources were considered adequate for the exploratory aim of this narrative review, which maps the scope of occupational therapy involvement in sensory stimulation rather than conducting a systematic review. This exclusion is acknowledged as a limitation.

Inclusion Criteria

Included studies involved adults (≥18 years) with TBI who were in comatose states (Glasgow Coma Scale (GCS) ≤8) and receiving care in acute care units, intensive care units (ICUs), or rehabilitation settings. The interventions included sensory stimulation therapies designed to promote arousal and awareness, involving auditory, visual, tactile, olfactory, gustatory, vestibular, or multimodal sensory inputs delivered repetitively and tailored to the patient's condition. Studies assessing changes in consciousness or neurological responsiveness using standardized outcome measures (e.g., GCS, Coma Recovery Scale-Revised (CRS-R), Richmond Agitation and Sedation Scale, Western Neuro-Sensory Stimulation Profile) were included.

Study Selection and Gap Identification

**Figure 1:**
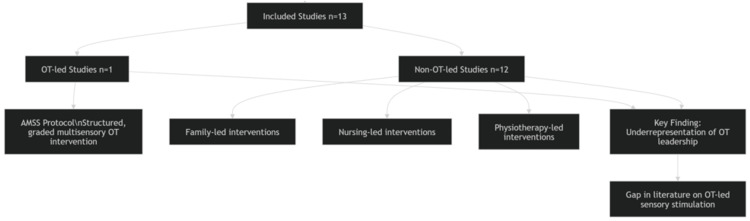
Distribution of included studies by provider type in sensory stimulation interventions OT: occupational therapist

A total of 13 studies met the inclusion criteria and were included in the final narrative review. A critical analysis of the provider role revealed a significant disparity: among these, only one study was explicitly led by OT. The remaining 12 studies focused primarily on interventions delivered by family members, nursing staff, or physiotherapists (Figure 1). This distribution highlights a significant gap in the literature regarding the specific leadership and expertise of occupational therapists in sensory stimulation protocols.

As this is a narrative review, the aim was to synthesize existing approaches rather than establish a standardized intervention protocol.

## Review

The hidden role of OTs in sensory stimulation protocols

OTs aim to enable participation in daily activities. Their expertise in sensory integration allows them to structure and grade stimuli to increase arousal without overstimulation. OTs select patient-specific stimuli, monitor behavioural responses (e.g., recognition, localization, and emotional reactions), and design individualized protocols including modality choice, intensity, duration, and scheduling. While the number of OT-specific studies was limited (n=1), the effect sizes and statistical significance reported in this study suggest potential benefits compared with non-specialized protocols [2].

Overview of included studies

This narrative review synthesized evidence from 13 clinical studies published within the last decade, investigating the efficacy of sensory stimulation interventions for comatose patients following TBI. The included studies employed various designs, including randomized controlled trials (RCTs), quasi-experimental studies, and case reports, with sample sizes ranging from 30 to 90 participants. Interventions varied from unimodal approaches (e.g., auditory or tactile stimulation) to multimodal protocols incorporating auditory, visual, tactile, olfactory, gustatory, and kinaesthetic stimuli.

Across all studies, statistically significant improvements in level of consciousness (LOC) were reported as measured by the GCS and/or CRS-R. However, variation in intervention delivery was observed depending on the professional background of providers, particularly in relation to structure, individualization, and integration of sensory modalities (Table 1). Of note, one occupational therapy-led study applied an adapted multimodal sensory stimulation (AMSS) protocol, which integrates multiple sensory channels in a structured, graded, and individualized manner, representing a more comprehensive example of OT-informed sensory integration within this evidence base.

**Table 1:** Overview of included studies on sensory stimulation in comatose TBI patients CST: coma stimulation technique; AMSS: adapted multimodal sensory stimulation; RCT: randomized controlled trial; OT: occupational therapist; TBI: traumatic brain injury; GCS: Glasgow Coma Scale; ROM: range of motion; HR: heart rate; RR: respiratory rate; BP: blood pressure; RLA: Rancho Los Amigos; WNSSP: Western Neuro-Sensory stimulation profile; CRS-R: Coma Recovery Scale-Revised; FOUR: Fall Outline of Unresponsiveness Score; DRS: Disability Rating Scale

Study reference	Design	Intervention Type	Provider	OT Involvement	Duration	Key Outcomes	OT-Relevant Elements
Sindhubhai et al., 2023 [6]	Pre-experimental	CST (Visual, Olfactory, Gustatory, Tactile, Auditory, Kinesthetic)	Nurses	NO	6 days	GCS: 6→10; CRS-R: 5→14	Multimodal; ROM exercises; structured protocol; once daily
YekeFallah et al., 2018 [7]	Clinical trial	Tactile hand stimulation	Nurses	NO	5 days	GCS increased; HR, BP, RR decreased	Wrist to fingertips; 5 minutes twice daily; gentle touch
Salehi et al., 2019 [8]	RCT	Foot reflexology / Tactile hand stimulation	Nurses	NO	5 days	Foot reflexology best: GCS 8.2→10.7	Touch therapy; mechanical effects; twice daily; 5-minute sessions
Moattari et al., 2016 [9]	RCT	Multimodal sensory (Auditory, Visual, Olfactory, Tactile)	Nurses/Family	NO	7 days	Family group: GCS 5.75→9.20; RLA & WNSSP improved	Family training; meaningful stimuli; twice daily; patient preferences considered
Salmani et al., 2017 [10]	RCT	Family-centered affective stimulation	Family members	NO	7 days	GCS: 5.3→9.1; CRS-R: 5.5→11.9	Emotional connection; meaningful interaction; kinaesthetic stimulation; 30-45-minute sessions
Ahmed et al., 2023 [11]	Quasi-experimental	Auditory + Tactile stimulation	Trained family	NO	14 days	GCS: 8.43→12.90; Reduced PAEs; Shorter ICU stay	Family training; structured protocol; simultaneous stimulation; 15 minutes/day
Shinde & Kanase, 2024 [12]	RCT	Rood's approach + Coma Stimulation	Physiotherapist	Partial: Neurophysiological technique	28 days	GCS: 3.6→13.7; CRS-R: 1.4→20.7	Rood's techniques used in OT; fast brushing, icing, vibration, tapping; multisensory
Hoseini et al., 2022 [13]	Triple-blind RCT	Auditory (Family voice + Music)	Nurses	NO	6 days	GCS: 7.35→10.25; RLA: 3.6→5.46	Familiar voices; positive memories; music; 15-minute twice daily
Yekefallah et al., 2020 [14]	Triple-blind RCT	Musical stimulation	Nurses	NO	7 days	GCS improved from day 3; 60-80 bpm music	Music therapy; 15 minutes/day; headphones; familiar music
Hoseinzadeh et al., 2018 [15]	Single-blind RCT	Organized Auditory Stimulation (Nurse's voice)	Nurses	NO	10 days	GCS: 6.05→11.85	Orientation content; place/time/identity; 10 min thrice daily
Wulandari et al. 2024 [16]	Case study	Auditory + Tactile	Trained family	NO	7 days	GCS: 8→14; FOUR: 9→14	Simultaneous stimulation; family training; 10 minutes/day; meaningful content
Naik & Vajaratkar, 2024 [17]	Quasi-experimental	AMSS (Acoustic, Olfactory, Gustatory, Visual, Tactile, Kinaesthetic, Proprioceptive)	OT	YES - OT-led	14 days	GCS: 6.73→12.20; CRS-R: 3.4→14.53; DRS: 23.26→15.13	Individualized meaningful stimuli; sensory integration; graded intervention; 2 hours/day; patient preferences
Chuaykarn & Jitpanya, 2017 [18]	Experimental	4-modality vs 5-modality sensory	Nurses	NO	14 days	4-modality superior: CRS-R higher	Auditory, Visual, Tactile, Kinesthetic; twice daily 30 minutes; structured

Efficacy of sensory stimulation across providers

Consistent with existing literature, the review confirms that structured sensory stimulation is effective in promoting arousal and neurological recovery in comatose TBI patients. Studies led by nursing staff reported significant gains in GCS scores, with improvements ranging from 2 to 5 points over intervention periods of one to four weeks [6-8]. Similarly, studies involving family members as intervention providers often yielded superior outcomes compared to standard nursing care, attributed to the emotional significance and familiarity of the stimuli [ 9-11]. Additionally, physiotherapy-led interventions utilizing neurophysiological facilitation techniques, such as Rood's approach, demonstrated improvements in arousal and motor responsiveness [12]. Despite these positive outcomes, a critical analysis of the intervention providers reveals a significant disparity in professional involvement.

Unimodal interventions, particularly those utilizing auditory pathways, have also demonstrated significant efficacy in promoting neurological arousal. For instance, a triple-blind randomized clinical study found that auditory sensory stimulation using familiar family voices combined with music significantly improved GCS and cognitive function scores from day 3 onward [13]. Similarly, another triple-blind randomized clinical study demonstrated that musical stimulation alone could significantly increase LOC from day 3 onward [14], while a single-blind randomized clinical study showed that organized auditory stimulation by a nurse's voice improved GCS means from 6.05 to 11.85 over 10 days [15]. These findings confirm that sensory input, regardless of modality, activates the Reticular Activating System (RAS) and promotes arousal. However, while the efficacy of the intervention is well-documented, the expertise required to design and grade these interventions remains underutilized.

In addition to the studies mentioned previously, a case study in Indonesia demonstrated that family-centered auditory and tactile stimulation, delivered by trained relatives for 10 minutes daily over seven days, improved GCS scores from 9 to 14 in a 20-year-old male with severe TBI [16]. This finding reinforces the value of structured, emotionally significant sensory input delivered by familiar persons.

The occupational therapy gap: provider disparity

Despite the established efficacy of sensory stimulation, this review identified a pronounced gap in OT leadership within this domain. Of the 13 studies analysed, only one randomized study was explicitly led by OT. The remaining studies were predominantly conducted by nursing staff (n=8) [6-9,13-15,18], family members (n=4) [9-11,16], or physiotherapists (n=1) [12]. This scarcity is notable given that sensory integration and the analysis of occupational performance are foundational competencies of occupational therapy practice. The dominance of nursing-led protocols often emphasizes physiological stability and routine care integration, whereas physiotherapy-led protocols prioritize motor facilitation. The limited representation of OT suggests an underutilization of specialized expertise in sensory processing and functional adaptation during the acute coma phase.

Unique contributions of OT-led interventions

The single OT-led study provides critical insights into the unique value proposition of occupational therapy in coma stimulation [17]. When compared to non-OT-led interventions, the OT-led protocol demonstrated three distinct advantages:

Measurement of Functional Outcomes

While most included studies utilized the GCS and CRS-R to measure arousal and neurological responsiveness, the OT-led study was the only one to incorporate the Disability Rating Scale (DRS). Results indicated a 35% reduction in disability scores (from 23.26 to 15.13) alongside improvements in consciousness. This distinction is important, as nursing and medical models primarily emphasize survival and arousal, whereas occupational therapy prioritizes functional recovery and engagement.

The reduction in DRS may reflect not only improved arousal but also emerging readiness for rehabilitation. By organizing and grading sensory input, OTs may enhance the integration of sensory information, supporting early attentional regulation, motor responsiveness, and cognitive engagement. In this sense, sensory stimulation can be understood as a functional bridge linking basic arousal to higher-level participation. The inclusion of the DRS, therefore, aligns with the OT paradigm of enabling occupation and suggests that OT-led interventions may better support the transition from arousal to functional recovery [17].

Individualization and Meaningfulness of Stimuli

The review highlights that family-led interventions often outperform standardized nursing protocols due to the "meaningfulness" of the stimuli. Family-led studies demonstrated that familiar voices, preferred music, and emotionally significant touch elicited stronger responses than generic stimuli [8,9,11]. The OT-led study operationalized this principle through AMSS, where stimuli were selected based on individual patient history, preferences, and occupational identity. This aligns with the client-centered practice framework central to occupational therapy. While family-led studies rely on natural family involvement, the OT model provides a structured, theoretically grounded method for selecting and grading these meaningful stimuli to maximize neural engagement without causing sensory overload [17]. This need for structured modulation is supported by nursing-led research; for instance, an experimental study in Thailand found that a streamlined four-modality program (auditory, visual, tactile, kinesthetic) resulted in significantly higher CRS-R scores after five days compared to a five-modality program and control group [18]. This suggests that carefully graded multimodal stimulation, avoiding potential safety risks or overload associated with excessive modalities, is more effective, reinforcing the need for OT expertise in designing safe, individualized protocols.

Sensory Integration and Grading

OTs possess specialized training in sensory integration theory, which emphasizes the grading of sensory input to match the patient's processing capacity. The OT-led protocol incorporated specific rest intervals and monitored for signs of sensory overload (e.g., physiological instability), adjusting frequency and intensity accordingly. In contrast, several nursing-led protocols utilized fixed durations and intensities regardless of patient response. The OT-led study achieved significant GCS and CRS-R improvements within a shorter duration (two weeks) compared to some 4-week nursing or physiotherapy protocols [17], suggesting that optimized, graded sensory input may offer greater time efficiency in the acute setting.

Multimodal vs. unimodal approaches

The review indicates a dose-response relationship favouring multimodal over unimodal stimulation. For instance, one study compared four-modality (auditory, visual, tactile, kinesthetic) versus five-modality (adding olfactory/gustatory) programs, finding that the four-modality group achieved significantly higher CRS-R scores, likely due to reduced aspiration risk and better tolerability [18]. In contrast, the OT-led study utilized a comprehensive seven-modality AMSS protocol (including vestibular and proprioceptive inputs), which was associated with substantial functional improvements in the included study [17]. This suggests that while increasing modalities carries risk, OT expertise in grading and sensory integration allows for the safe delivery of complex multimodal protocols. Conversely, while unimodal interventions like music or tactile stimulation were effective, their impact on cognitive recovery (CRS-R/DRS) was generally less profound than multimodal approaches.

Comparative analysis of intervention models

Comparative outcomes across provider types are summarized in Tables 2, 3. While physiotherapy-led interventions showed high absolute gains in GCS, this was achieved over a longer duration (four weeks) using neurophysiological facilitation aimed primarily at motor arousal. Nursing-led interventions demonstrated consistency in improving physiological stability (e.g., heart rate, blood pressure). However, the OT-led intervention uniquely bridged these domains by targeting arousal and functional disability simultaneously. This suggests that while nursing care ensures safety and physiotherapy facilitates motor pathways, occupational therapy integrates these elements to promote meaningful neurological reorganization oriented toward functional recovery. These findings highlight the importance of including occupational therapy as a core component of sensory stimulation protocols in the rehabilitation of comatose TBI patients.

**Table 2:** Distinctive features of OT-led graded stimulation versus standard care protocols OT: occupational therapist

Feature	Standard Stimulation (Nurse/Family)	Graded Stimulation (OT-Led)
Approach	Passive / Fixed	Active / Adaptive
Goal	General Arousal	Targeted Neuroplasticity
Monitoring	Completion of task	Behavioral & Autonomic response
Complexity	Single or set Multimodal	graded or sequentially ordered
Sources	[6-16,18]	[17]

**Table 3:** Comparative summary of provider personnel and clinical impact OT: occupational therapist; LOC: level of consciousness; GCS: Glasgow Coma Scale; CRS-R: Coma Recovery Scale-Revised; DRS: Disability Rating Scale

Feature	OT Study	Nurse/Family Studies
Personnel	OTs applying facilitation techniques	Nurses or trained family members
Sensory Modalities	Multimodal	limited modalities
Structure	Early, repetitive, individualized programs	Often fixed schedule, fewer modalities, less patient-specific
Outcome Magnitude	Significant improvements in GCS, CRS-R, DRS; faster recovery	Improvements noted but smaller effect sizes; slower recovery
Functional Impact	Includes arousal, responsiveness, and functional recovery	Primarily LOC and basic cognitive gains
Clinical Implication	OT facilitation amplifies the effect of sensory stimulation, providing structured neurorehabilitation	Effective but may not maximize recovery potential
Source	[17]	[6-16,18]

Why OT-led interventions demonstrate enhanced results

The OT-led study by Naik and Vajaratkar (2024) [17] demonstrated enhanced results compared to interventions utilizing only Rood's technique or other non-OT approaches for several evidence-based reasons. First, it is the only study among the 13 reviewed that measured functional disability outcomes using the DRS, showing a 35% reduction in disability (from 23.26 to 15.13), which aligns with occupational therapy's core focus on functional recovery beyond mere arousal. Second, the intervention employed individually adapted, meaningful stimuli based on each patient's preferences, history, and occupational identity, a hallmark of client-centered occupational therapy practice, whereas Rood's approach, while neurophysiologically sound, typically applies standardized facilitatory techniques without the same degree of personalization. Third, the OT-led protocol achieved significant improvements in consciousness (GCS: +5.47; CRS-R: +11.13) within only two weeks of intervention, suggesting greater time efficiency potentially attributable to the integration of sensory integration principles, graded stimulation, and prevention of sensory overload-competencies central to occupational therapy training. Finally, the large effect sizes reported (t = −10.20 for CRS-R) indicate that when sensory stimulation is delivered within an occupation-based, theoretically grounded framework, neural engagement and recovery may be optimized. While Rood's technique remains valuable for neurophysiological facilitation, the occupational therapy model extends beyond arousal to promote meaningful participation, functional re-engagement, and continuity of care outcomes that are essential for long-term recovery in TBI [17].

Limitations and future directions

While this review provides evidence for the benefits of sensory stimulation, several methodological limitations across the included studies must be acknowledged. Primarily, most studies utilized small sample sizes, which limits the generalizability of the findings to broader TBI populations. Additionally, the majority of research, including the OT-led study, employed short follow-up periods ranging from two to four weeks. This brevity prevents a comprehensive assessment of long-term functional independence and sustained recovery. Furthermore, intervention protocols varied widely in terms of modalities, duration, frequency, and personnel. In addition, substantial heterogeneity in intervention dosage, sensory modalities, and outcome measures limits the ability to define a standardized or reproducible OT-led protocol based on the current evidence base. This heterogeneity makes direct comparisons between studies difficult and complicates the synthesis of evidence regarding optimal stimulation parameters.

Specific limitations exist regarding the evidence base for occupational therapy within this field. Currently, the literature is dominated by studies involving nurses or family members, with limited examination of multimodal sensory stimulation led by OTs. Notably, the evidence base for OT-led sensory stimulation is currently limited to a single quasi-experimental study [17]. While the effect sizes reported in this study are large and statistically significant, the lack of multiple randomized controlled trials led by OTs restricts the generalizability of these findings. Compounding this issue is the absence of manualized, OT-specific protocols in the existing literature. This critical gap hinders the ability to replicate interventions and validate the distinct role of OT in neurocritical care.

Collectively, these limitations underscore the urgent need for methodological rigor in future research. To determine the full impact of multimodal sensory stimulation on recovery in comatose TBI patients, larger and standardized OT-led trials are necessary. Future studies should prioritize the development of manualized protocols to ensure consistency and allow for meaningful comparison across settings. By addressing these gaps, researchers can better establish the efficacy of occupational therapy interventions and integrate them as a standard of care for disorders of consciousness.

Implications for practice

The evidence synthesized in this review supports the integration of OTs into early coma management teams. Beyond direct intervention, OTs play a critical role as educators and consultants within the multidisciplinary team. Given that sensory stimulation occurs across the 24-hour care cycle, OTs should train family members and nursing staff to deliver graded, structured, and patient-specific stimulation, including guidance on timing, intensity, and monitoring responses.

Positioning the OT as a “sensory stimulation coordinator” promotes consistency and continuity of care by embedding stimulation strategies into routine interactions and aligning them with functional goals. Accordingly, hospital and ICU protocols should mandate early OT referral for comatose TBI patients to optimize sensory input and support the transition to functional rehabilitation.

## Conclusions

Sensory stimulation remains a widely used and supported intervention for patients with TBI and disorders of consciousness, regardless of provider type. This review highlights that occupational therapy is currently underrepresented within this area of practice. Available evidence suggests that occupational therapy-led approaches may contribute to more individualized, graded, and functionally oriented sensory stimulation, with one study demonstrating promising improvements in consciousness (GCS and CRS-R) and functional outcomes (DRS) within a shorter intervention period. However, given that these findings are derived from a single OT-led study among a heterogeneous body of literature, they should be interpreted as preliminary and hypothesis-generating rather than evidence of superiority. Instead, they indicate a potential role for occupational therapy in enhancing the structure and functional relevance of sensory stimulation interventions within early neurorehabilitation.

Future research should prioritize the development and validation of standardized OT-led sensory stimulation protocols, including clear grading frameworks and outcome measures that extend beyond arousal to functional recovery. Strengthening the evidence base through well-designed trials will be essential to define the precise role of occupational therapy in neurocritical care settings. In addition, greater integration of OTs within multidisciplinary ICU teams, alongside structured training for staff and families, may support more consistent delivery of individualized and graded sensory stimulation and improve continuity of care for this patient population.
